# The association of pre-photorefractive keratectomy Schirmer-1 test value with postoperative corneal epithelial thickness, ocular surface discomfort, and visual acuity

**DOI:** 10.5935/0004-2749.2023-0049

**Published:** 2024-03-05

**Authors:** İrfan Botan Güneş, Hakan Öztürk, Bediz Özen

**Affiliations:** 1 Department of Ophthalmology, Medicalpark Kocaeli Hospital, Kocaeli Health and Technology University, Kocaeli, Turkey; 2 Department of Ophthalmology, Tepecik Training and Research Hospital, University of Health Sciences, Izmir, Turkey

**Keywords:** Epithelium, corneal, Cornea, Photorefractive keratectomy, Schirmer test, Visual acuity

## Abstract

**Purpose:**

To investigate the association of pre-photorefractive keratectomy Schirmer-1 test value with post-photorefractive keratectomy central corneal epithelial thickness, ocular surface disease index score, and uncorrected distance visual acuity.

**Methods:**

Patients were categorized according to preoperative Schirmer-1 value: the normal Schirmer Group (n=54; Schirmer-1 test value, >10 mm) and the low Schirmer Group (n=52; Schirmer-1 test value, between 6 and 10 mm). We analyzed ablation depth, visual acuity, result of Schirmer-1 test (with anesthesia), tear film break-up time, ocular surface disease index score, central corneal epithelial thickness, and spherical equivalent refraction.

**Results:**

We found significant differences between the groups in Schirmer-1 test value, tear film break-up time, and ocular surface disease index score, both preoperatively and postoperatively (p<0.001). The preoperative central corneal epithelial thicknesses of the two groups were similar (p>0.05). After photorefractive keratectomy, the Schirmer-1 test value and spherical equivalent refraction decreased in both groups (p<0.05), and ocular surface disease index scores and central corneal epithelial thickness values increased in the low Schirmer Group (p<0.001) but not in the normal Schirmer Group (p>0.05). The postoperative central corneal epithelial thicknesses of the low Schirmer Group were significantly higher than those of the normal Schirmer Group (p<0.001). Postoperative uncorrected distance visual acuity did not differ significantly between the two groups (p>0.05).

**Conclusions:**

In patients with low Schirmer-1 test values before photorefractive keratectomy, the corneal epithelium thickened and ocular surface complaints increased during the postoperative period. However, changes in the corneal epithelium did not affect the postoperative uncorrected distance visual acuity. To reduce postoperative problems on the ocular surface in these patients, we recommend that dry eye be treated before photorefractive keratectomy.

## INTRODUCTION

Photorefractive keratectomy (PRK) is one of the most common refractive laser procedures today^(1)^. In this procedure, an excimer laser breaks down the molecular organic bonds in sensitive structures, such as epithelial and stromal layers of the cornea, through a photochemical reaction. With the ablative effect, the cornea can be reshaped to correct refractive errors^(2,3,4)^. During PRK, epithelial removal and the ablation process may damage the free nerve endings in the epithelium, the sub-basal nerve plexus, and the deeper stromal nerves, and such damage decreases corneal sensation^(3,4)^. Impaired corneal sensation may result in a reduction in tear secretion^(3,4,5)^. The normal tear film layer has important roles both in the healing process of the epithelium and stroma and in the patient’s comfort and post-PRK satisfaction^(5)^. Therefore, tear function status should be assessed in patients before they undergo PRK^(2,5)^.

Although tear flow can be assessed by more specific tests, the Schirmer-1 test is easily applicable, available, inexpensive, and practical for the evaluation of tear secretion^(5,6)^. The accuracy of the Schirmer test could be increased with the use of a topical anesthetic and closing of the eye^(5)^. For assessing tear film stability, tear film break-up time (TBUT) is the measure most frequently used in clinical practice^(6)^. The ocular surface disease index (OSDI) is the most widely used questionnaire for assessing patients’ complaints about the ocular surface, such as visual disturbance or visual function, the frequency of symptoms, and environmental triggers^(6)^.

The corneal epithelium protects the eye against external causes of injury. It also plays an important role in maintaining high optical quality^(7)^. Moreover, the corneal epithelium contributes 0.85 diopters to the refractive power of the cornea in a 3.6 mm diameter zone^(8)^. PRK may cause injury and structural changes in the corneal epithelium^(2,9)^. The changes in epithelial thickness may affect corneal refraction^(9,10)^; therefore, corneal epithelial thickness should be measured in patients before they undergo PRK^(10)^. Optical coherence tomography (OCT) is a noncontact and noninvasive imaging method of measuring corneal epithelial thickness^(8)^. According to the literature, OCT has good reproducibility in such measurement^(11,12)^.

The effect of relative tear deficiency on the results of refractive laser surgery and its relation to postoperative ocular surface complaints are topics of interest to ophthalmologists. Therefore, we aimed to investigate the association of pre-PRK Schirmer-1 test value with post-PRK central corneal epithelial thickness (CCET), OSDI score, and uncorrected distance visual acuity (UDVA).

## METHODS

This study was approved by the Istinye University Ethics Committee and was conducted in accordance with the ethical principles of the Helsinki declaration. Detailed information about the study and the risks was given to participants, all of whom provided informed consent to participate. This study initially consisted of 148 patients who underwent PRK for myopia by one surgeon. Only the right eyes of all participants were studied. The exclusion criteria were allergic conjunctivitis, corneal disease, glaucoma, systemic disease such as diabetes and connective tissue disease, chronic topical or systemic drug use, a history of previous ocular surgery, and the use of contact lenses. Patients with clinically significant dry eye (grade 3 or worse according to the severity classification of the Dry Eye WorkShop^(13)^) at the preoperative examination and patients who did not complete follow-up examinations were also excluded from the study. The final study population included 106 patients.

We documented the detailed drug and medical histories of the patients. UDVA and corrected distance visual acuity (CDVA) were recorded as logarithm of the minimum angle of resolution (logMAR) values. As in similar studies, CDVA for the preoperative period and UDVA for the postoperative period were evaluated^(14,15)^. Detailed anterior segment and fundus examinations of the patients were performed with a slit-lamp biomicroscope and a 90-diopter lens. Intraocular pressure was measured with noncontact tonometry (CT-11P noncontact tonometer; Topcon, Tokyo, Japan). Manifest and cycloplegic refraction measurements were made with the KR1 Auto Kerato-Refractometer (Topcon). Topical 1% cyclopentolate hydrochloride was used to induce cycloplegia. Three days before and 3 months after PRK, we performed the Schirmer-1 test (with anesthetic) and OSDI scoring and measured TBUT, CCET, and spherical equivalent refraction (SER). In the Schirmer-1 test (with anesthetic), the standard Schirmer test paper was placed on the outer third of the edge of the lower eyelid after the administration of topical 0.5% proparacaine hydrochloride. During this test, the patients kept their eyes closed. The amount of wetting on the paper was measured after 5 minutes. To measure TBUT, fluorescein-dyed paper was moistened with saline solution; then this paper was placed against the lower conjunctival fornix and remained there until the fluorescein was completely dispersed on the corneal surface. The time (in seconds) from the last blink to the first break of the tear film was recorded. The Schirmer-1 test and TBUT measurement were performed in a room with relatively constant temperature and humidity. The total OSDI score was calculated with the formula


OSDI=([sum of the scores of all questions answered]25)/(total number of questions answered)

**Figure 1:**
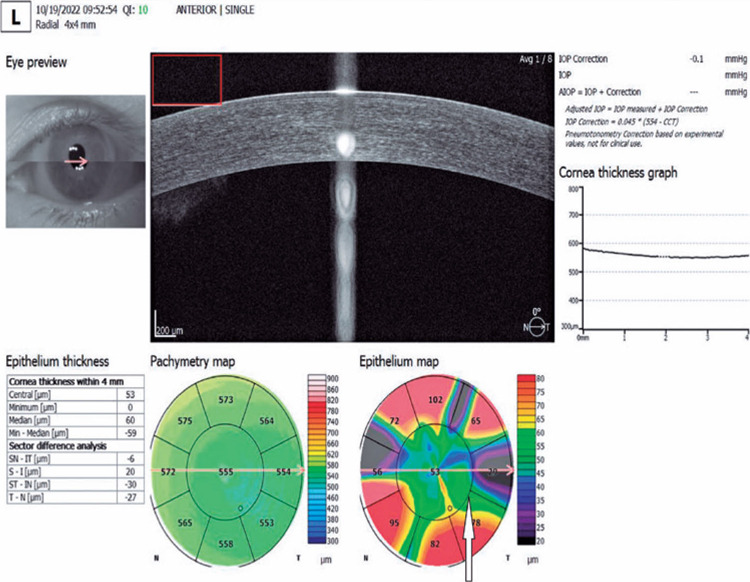
Corneal epithelial thickness mapping with spectral domain optimal optical coherence tomography. The central zone ring is indicated by the white arrow. Mean corneal epithelial thickness in the central zone was used in the analysis of measurements. Unit: micrometers.

The results (scores of 0-100) were evaluated^(6)^. To construct maps of participants’ corneal epithelial thicknesses, we used the corneal acquisition mode of the spectral domain OCT device (REVO NX 130; Optopol Technology Sp z o.o, Zawiercie, Poland). The thicknesses of the corneal epithelium were examined in a circular area with a 4-mm diameter. The corneal epithelial thickness of the central area, with a 2-mm diameter, was defined as the CCET (Figure 1). The SER was calculated as sphere power + ½ cylinder power. All measurements were made by the same specialist, and the average value of three measurements was recorded. The 106 participants were assigned to one of two groups according to preo-perative Schirmer-1 test values: 54 with preoperative Schirmer-1 test values of >10 mm (the normal Schirmer Group) and 52 with preoperative Schirmer-1 test values between 6 and 10 mm (the low Schirmer Group).

All surgical procedures were performed with the VISX STAR S4 IR™ Excimer Laser (VISX, Incorporated, Santa Clara, CA, USA) by one surgeon (IBG). PRK was performed in patients with myopia up to 4.50 diopters and astigmatism up to 0.50 diopters. Because the astigmatism values were low before PRK, the procedure was applied mainly to correct myopia. Before the procedure, the patients’ eyelids were cleaned with 5% betadine solution. After their eyes were closed with a drape, topical 0.5% proparacaine hydrochloride solution was placed on the eyelids. After a 20% alcohol solution was applied to the cornea for 15 seconds with an 8-mm diameter corneal trephine, the corneal epithelium was debrided with a triangular sponge. After epithelial debridement, stromal ablation was performed with a laser at a frequency of 400 Hz and a wavelength of 193 nm in the 6.5-mm ablation zone. Then a 0.02% mitomycin C-soaked sponge was applied to the ablation site for 5 seconds. After washing with a balanced salt solution, bandage contact lenses were applied to the eye as the last step of the operation. In the postoperative period, 0.5% moxifloxacin drops were used four times a day for 1 week; 0.1% dexamethasone drops, four times a day for 1 month; and preservative-free artificial tear drops (Artelac Advanced; Bausch + Lomb, Laval, QC, Canada), every 2 hours for 1 month. The patients were routinely examined on the third and tenth days and 1 and 3 months after PRK. When corneal epithelization was complete, typically between 3 and 10 days after the procedure, the bandage contact lenses were removed.

We used SPSS 15.0 for Windows (IBM SPSS Inc., Chicago, IL, USA) to perform all statistical analyses. Values for descriptive characteristics were calculated as means ± standard deviations. After a test of normality (Kolmogorov-Smirnov test), we performed independent and paired-samples t tests for homogeneous results and the Mann-Whitney U test for heterogenous results. For nominal data, we performed the chi-square test. Pearson correlation analysis was used to determine the relationship between the degree of myopia and the postoperative Schirmer-1 test value. The results were analyzed with 95% confidence intervals, and p-values of <0.05 were considered statistically significant.

## RESULTS

We studied one eye in each of the 54 participants in the normal Schirmer Group and in each of the 52 participants in the low Schirmer Group. Demographic data (age and gender), ablation depth, and visual acuity of the groups are listed in Table 1. We found no statistically significant differences between the normal and low Schirmer Groups in age (p=0.34) or gender distribution (p=0.396). Ablation depth, preoperative CDVA, postoperative UDVA, preoperative CCET, and preoperative and postoperative SER of the normal Schirmer Group were similar to those of the low Schirmer Group (each p>0.05). We also found no statistically significant differences between the two groups in astigmatism value, either preoperatively (p=0.596) or postoperatively (p=0.584).

**Table 1:** Demographic data, ablation depth, and visual acuity of the groups

Characteristic	Normal Schirmer group (n=54)	Low Schirmer group (n=52)	p-value*
Age (years)	26.35 ± 4.05 (21–36)	27.25 ± 4.42 (20–36)	0.346^a^
Gender (male/female)	32/22	28/24	0.396^b^
Ablation depth (micrometers)	46.68 ±1.52 (42–54)	46.54 ± 1.54(43–52)	0.598^a^
Preoperative CDVA (logMAR)	0.007 ± 0.02 (0–0.1)	0.009 ± 0.03 (0–0.1)	0.763^c^
Postoperative UDVA (logMAR)	0.009 ± 0.29 (0–0.1)	0.004 ± 0.02 (0–0.1)	0.370^c^

Values of descriptive characteristics are listed as means ± standard deviations (range).

CDVA= corrected distance visual acuity; logMAR= logarithm of the minimum angle of resolution; UDVA= uncorrected distance visual acuity.

^a^Independent *t* test. ^b^Chi-square test. ^c^Mann-Whitney *U* test.

p-values of <0.05 were statistically significant.

We did find significant differences between the groups in Schirmer-1 test values, TBUT, and OSDI score both preoperatively and postoperatively (each p<0.001). Postoperative CCET was significantly greater in the low Schirmer Group than in the normal Schirmer Group (p<0.001). Ocular characteristics of the groups are listed in Table 2.

**Table 2:** Ocular characteristics of the two groups Values of descriptive characteristics are listed as means ± standard deviations (range)

Characteristic	Before PRK	Three months after PRK	p-value
Schirmer-1 test value (millimeters)	7.13 ± 1.09 (6–9)	6.61 ± 1.46(2–9)	<0.001^a^
TBUT (seconds)	7.06 ± 1.03 (6–9)	6.97 ± 1.77(4–12)	0.697^a^
OSDI score	29.82 ± 13.65 (4.16–56.16)	34.93 ± 13.25(2.08–58.24)	<0.001^a^
CCET (micrometers)	59.27 ± 2.00 (55–64)	63.34 ± 3.64 (55–69)	<0.001^a^
SER (diopters)	3.32 ± 0.59 (-1.50 to -4.00)	0.08 ± 0.17(0 to -0.75)	<0.001^a^
Astigmatism (diopters)	0.08 ± 0.04 (0 to -0.50)	0.07 ± 0.06 (0 to -0.50)	0.602^a^

TBUT= tear film break-up time; OSDI= ocular surface disease index; CCET= central corneal epithelial thickness; SE= spherical equivalent refraction.

a Independent *t* test; p values of <0.05 were statistically significant.

In the normal Schirmer Group, Schirmer-1 test values and SER were significantly lower by the third month after PRK than before PRK (p=0.01 and p<0.001, respectively), and TBUT, OSDI score, CCET, and astigmatism values after PRK were similar to those before PRK (each p>0.05). Ocular data before and after PRK for the normal Schirmer Group are listed in Table 3. In the low Schirmer Group, Schirmer-1 test values and SER were significantly lower by the third month after PRK than in before PRK (p<0.001), whereas OSDI score and CCET value increased significantly (p<0.001); TBUT and astigmatism values after PRK were similar to those before PRK (p>0.05). Ocular data before and after PRK for the low Schirmer Group are listed in Table 4. We found no significant correlation between preoperative degrees of myopia and postoperative Schirmer-1 test values in the whole sample (r=0.102, p=0.298).

**Table 3:** Ocular data before and after PRK for the normal Schirmer group

Characteristic	Before PRK	Three months after PRK	p-value
Schirmer-1 test value (millimeters)	23.57 ± 5.83 (12–35)	22.81 ± 5.88 (11–35)	0.01^a^
TBUT (seconds)	14.12 ± 2.51 (11–22)	14.22 ± 2.20 (8–21)	0.688^a^
OSDI score	5.00 ± 4.25 (0–12.48)	5.08 ± 4.26 (0–16.64)	0.926^a^
CCET (micrometes)	58.53 ± 2.41 (54–64)	58.59 ± 2.81 (55–67)	0.768^a^
SER (diopters)	3.02 ± 0.51 (1.25 to -3.75)	0.06 ± 0.16 (0 to -0.75)	<0.001^a^
Astigmatism (diopters)	0.07 ± 0.05 (0 to -0.50)	0.06 ± 0.03 (0 to -0.50)	0.579^a^

Values of descriptive characteristics are listed as means ± standard deviations (range).

PRK= photorefractive keratectomy; TBUT= tear film break-up time; OSDI= ocular surface disease index; CCET= central corneal epithelial thickness; SER= spherical equivalent refraction.

a Paired-sample *t* test; p-values of <0.05 were statistically significant.

**Table 4:** Ocular data before and after PRK for the low Schirmer group

Characteristic	Before PRK	Three months after PRK	p-value
Schirmer-1 test value (millimeters)	7.13 ± 1.09 (6–9)	6.61 ± 1.46 (2–9)	<0.001^a^
TBUT (seconds)	7.06 ± 1.03 (6–9)	6.97 ± 1.77 (4–12)	0.697^a^
OSDI score	29.82 ± 13.65 (4.16–56.16)	34.93 ± 13.25 (2.08–58.24)	<0.001^a^
CCET (micrometers)	59.27 ± 2.00 (55–64)	63.34 ± 3.64 (55–69)	<0.001^a^
SER (diopters)	3.32 ± 0.59 (1.50 to 4.00)	0.08 ± 0.17 (0 to 0.75)	<0.001^a^
Astigmatism (diopters)	0.08 ± 0.04 (0 to 0.50)	0.07 ± 0.06 (0 to 0.50)	0.602^a^

Values of descriptive characteristics are listed as means ± standard deviations (range).

PRK= photorefractive keratectomy; TBUT= tear film break-up time; OSDI= ocular surface disease index; CCE= central corneal epithelial thickness; SE= spherical equivalent refraction.

a Paired-sample *t* test; p-values of <0.05 were statistically significant.

## DISCUSSION

Because PRK is an effective, reliable, and relatively common procedure in the treatment of mild to moderate ametropia, studies of PRK are of interest to ophthalmologists^(1,2)^. Different results regarding the effect of PRK procedure on Schirmer value has been reported^(4,16,17)^. The Schirmer test was performed without an anesthetic by Ozdamar et al.^(4)^ but with an anesthetic by Hong et al.^(16)^. On the other hand, Siganos et al.^(17)^ applied the Schirmer test first without and then with an anesthetic. In our study, we performed the Schirmer test measurements with an anesthetic to inhibit reflex tear secretion. According to the literature, the accuracy of the Schirmer test is increased by the use of a topical anesthetic^(5)^. Hong et al. found no change in Schirmer value after PRK; they attributed the absence of change to the presence of reflex tearing in the first 1-2 postoperative months^(16)^. In patients who underwent unilateral PRK, however, Ozdamar et al. found that Schirmer values in operated eyes were significantly lower than those of the nonoperated contralateral eyes^(4)^. Siganos et al. showed that Schirmer values were reduced after PRK^(17)^. In assessing tear secretion, we similarly found that Schirmer-1 test values decreased significantly in both the normal and low Schirmer Groups after PRK. Injury of the corneal sensory nerves during PRK may result in reduced sensory feedback to the lacrimal gland and decreased tear secretion^(3,4,5,18)^.

Conflicting effects of PRK on TBUT value have been reported^(16,19)^. Hong et al. found a reduction in TBUT after PRK; they speculated that ocular surface irregularity, especially soon after PRK, caused instability in the tear film layer^(16)^. In contrast, Nejima et al. found that TBUT did not change significantly after PRK^(19)^. We similarly detected no change in TBUT 3 months after PRK in both groups; we hypothesized that the reason might be related to the fact that the PRK procedure did not have a direct effect on meibomian gland function. Therefore, tear evaporation may not have been affected in our study.

We found that OSDI scores increased in the low Schirmer Group after PRK, whereas they did not vary significantly in the normal Schirmer Group. Previous studies showed that OSDI scores increased after surgery with an excimer laser^(5,20,21)^. In addition to the increase in OSDI scores in those studies, the TBUTs decreased significantly. In our study, the reason why OSDI scores in the normal Schirmer Group did not change might be related to the fact that TBUTs were mostly within the normal range after PRK. The significant increase in OSDI scores in the low Schirmer Group might be associated with the fact that TBUTs were mostly below normal after PRK. Furthermore, a much more significant reduction in Schirmer-1 values after PRK in the low Schirmer Group might have contributed to the increase in OSDI scores.

According to the literature, damage to corneal nerves, epithelial layers, and stromal layers may be caused by PRK^(2,3,9)^. The corneal epithelium, which is a layer of stratified nonkeratinized squamous epithelium, has a refractive power of approximately 0.85 diopters in a 3.6 mm diameter zone, and it protects the cornea from external causes of injury^(7,8)^. The changes in corneal epithelial thickness after refractive surgery may affect the patient’s final visual acuity as a result of changes in the refractive power^(7,8,9,10)^. In our study, the mean CCET increased significantly in the low Schirmer Group after PRK but did not differ significantly in the normal Schirmer Group.

In PRK for myopia, the center of the cornea is flattened, which reduces its refractive power^(9)^. The corneal epithelium over the flattened stroma can undergo gradual hyperplasia to partially compensate for stromal tissue loss^(9,10,22)^. This compensatory response of the epithelium may cause central epithelial thickening after PRK^(7,9,10)^. Moshirfar et al. theorized that epithelial compensation might lead to optical regression after laser refractive surgery^(9)^. Corneal epithelial thickening, approximately 5 µm in the first month after corneal refractive surgery, was reported to be a compensatory response to stromal irregularities ^(10)^. Surgery-related trauma in the corneal nerves sometimes results in neurotrophic epitheliopathy, dry eye syndrome, or persistent epithelial defects^(3)^. After epithelial damage in the cornea, cytokines such as insulin-like growth factor, transforming growth factor β, and keratinocyte growth factor can cause an increase in mitotic activity for epithelial stem cells^(9,23)^. Previous studies showed that cytokine and chemokine levels increased in the ocular surface and tear content of patients with dry eye^(24,25,26)^. Moreover, Kanellopoulos et al. found that dry eye could affect the corneal epithelial thickness^(27)^.

In our study, we hypothesized that the significant increase in CCET after PRK in the low Schirmer Group might be associated with higher cytokine levels on the ocular surface than in the normal Schirmer Group. On the other hand, Zhou and Stojanovic demonstrated that corneal epithelial thickness in healthy eyes with astigmatism values between 2.0 and 6.4 diopters showed insignificant deviations in distribution along the steepest meridian and the flattest meridian^(28)^. Sedaghat et al. reported that corneal epithelial thickness did not differ statistically between patients with low (<2 diopters) and moderate (2-4 diopters) astigmatism values in the central 2-mm area and along the different meridians^(29)^. We similarly hypothesized that astigmatism value was not associated with epithelial thickness change because both groups had low astigmatism values and CCET measurements were made from the central area with a 2-mm diameter.

Although we found a difference between the groups in mean CCET value 3 months after PRK, the UDVA and spherical equivalent values did not differ significantly between the groups. Therefore, we hypothesized that this level of difference in CCET 3 months after PRK did not affect the post-PRK UDVA and spherical equivalent values. Ivarsen et al. similarly reported that the increase in corneal epithelial thickness after PRK and LASIK had no refractive effect^(22)^.

The main limitation of our study was that cytokine levels on the ocular surface could not be measured. We determined that in the normal Schirmer Group, the OSDI score and CCET value did not change after PRK. On the other hand, we found that in patients with low Schirmer-1 test values before PRK, the corneal epithelium thickened and ocular surface complaints increased after PRK. However, in our study, this level of change in corneal epithelium did not affect post-PRK UDVA. Although we detected a statistically significant difference in dry eye scores between the two groups, we hypothesized that this level of difference would not affect results in clinical practice. Furthermore, we speculated that this decrease in dry eye scores in the low Schirmer Group could not preclude surgery, and PRK could be applied successfully and safely to patients with mild and moderate dry eye. On the other hand, these changes in dry eye scores should be kept in mind in preoperative evaluation of patients with severe dry eye and very low Schirmer values. For reducing postoperative adverse effects related to the ocular surface in patients with dry eye, we recommend that dry eye be treated before PRK.
